# Supplementary material for: TastepepAI: An artificial intelligence platform for taste peptide de novo design
Source: PLoS Comput Biol. 2025 Oct 16;21(10):e1013602. doi: 10.1371/journal.pcbi.1013602 (PMC12543283; doi:10.1371/journal.pcbi.1013602)
Supplement: S1 File — (ZIP) [file pcbi.1013602.s006.zip › Supplementary Data 2/TaPep12 HPLC.doc]

**HPLC REPORT**

| Sample: | FCL-NJP93313 TaPep12 YV-5 | | | Analyzed date: | 2024-9-25 |
| --- | --- | --- | --- | --- | --- |
| Analyst: | WJ | | | Reconstitution: | 100%H_2_O |
| Lot. No.: | NJP93313-1172216 | | | | |
| Column: | 4.6×250mm,Venusil MP C18-5 | | | | |
| Solvent A | A: 0.1% Trifluoroacetic Acid in 100% Acetonitrile | | | | |
| Solvent B | B: 0.1% Trifluoroacetic Acid in 100% Water | | | | |
| Gradient: |  | A | B |  | |
|  | 0.0min | 5% | 95% |  | |
|  | 25.0min | 30% | 70% |  | |
|  | 25.1min | 100% | 0% |  | |
|  | 30.0min | Stop | |  | |
| Volume: | 5μl | | |  | |
| Wavelength: | 220nm | | |  | |
| Flow rate: | 1.0ml/min | | |  | |

─────────────────────────────────────────────────────────────

Rank	Time	Conc. Area	Height

────────────────────────────────────────────────────────────

1	7.838	1.4825	189932	39808

2	8.018	96.6780	12386337	973706

3	8.338	1.2275	157270	36393

4	9.496	0.6120	78415	10055

────────────────────────────────────────────────────────────

Total	100	12811954	1059962
